# Obstetrics

**Published:** 1893-06

**Authors:** L. M. Griffiths


### OBSTETRICS.

In the Report on Obstetrics in December last, I gave a summary of the history of symphysiotomy up to that date. Since then many cases have been reported.[1] In the chorus of enthusiasm which accompanies a new or revived operation it is well that there should be a warning note, and this has been sounded by more than one surgeon.

Dr. Smyly's case, which I then mentioned, has now been reported in full.[2] As soon as the cartilage was divided the bones sprang apart, tearing the soft parts and the urethra. Although the urethra was sutured at the time, it did not completely heal, and a subsequent operation was required to cure the resulting incontinence.

Dr. Henry C. Coe reports[3] a case in which death took place two months after the operation. Firm union had taken place at the site of the pubic section. Acute mania had quickly supervened upon the operation. At the autopsy no cerebral lesion could be discovered, although partial hemiplegia had been present. Dr. Coe considers that there will always be much difficulty in deciding whether a skilful operator can deliver through a moderately contracted pelvis; and we should therefore be slow in adopting the operation, through fear of being accused of practising it unnecessarily.

In the discussion[4] which followed Dr. Coe's introduction of the subject in the New York Academy of Medicine, the general conclusion was that, if possible, the danger should be avoided of performing symphysiotomy in cases in which it would be impossible by the method to extract a living child, or in which Cæsarian section should be the operation of election. Dr. Edward P. Davis reported a case in which death occurred, from alcoholic pneumonia, seventy-two hours after the operation.

Dr. Henry J. Garrigues, in a dispassionate essay[5] on symphysiotomy, concludes that it is an operation not to be undertaken by anybody who is not familiar with the way of measuring a pelvis, or judging of the size and of other peculiarities of a child *in utero*, who has not the necessary instruments for this and other operations, and who is not capable of performing all other obstetric operations, inclusive of Cæsarian section and Porro's operation. He had a successful case which he operated upon in a common tenement-house. Recovery was hindered by serious puerperal infection.

Dr. W. T. Lusk reports[6] a case in which both mother and child died. The operation was performed under very unpropi-

[1] See current numbers of *Index Medicus*.
[2] *Brit. Med. Journ.*, Ap. 29, 1893. [3] *Med. Rec.*, Ap. 22, 1893.
[4] *Amer. Journ. Obst.*, May, 1893.
[5] *Amer. Journ. Med. Sci.*, Mar., Ap., 1893.
[6] *Ibid.*, Ap., 1893.

tious circumstances, as much damage to the soft parts, including the anterior vaginal wall and the cervix, had been caused by attempts to effect delivery. The mother died from collapse a few hours after the birth of the child.

Prof. A. Pinard, lecturing[1] on December 7th, was able to show all the thirteen mothers upon whom symphysiotomy had been performed, in the Clinique Baudelocque between February 4th and November 14th, 1892. Their general condition left nothing to be desired. In each the pelvis was as firm as before the operation, and there was no trouble in micturating or in standing. All thirteen children were born alive, but three died. Prof. Pinard differs from the American authorities in his operative procedure, which he describes with much detail. He considers that symphysiotomy ought to occupy a foremost place amongst obstetrical operations, because it is essentially a conservative operation. It is his opinion that we are for ever rid of the nightmare of an embryotomy practised on a living fœtus, and that the Cæsarian operation in cases of contracted pelvis will be scarcely ever necessary.

Prof. Pinard does not feel himself yet in a position to speak positively on the relation between symphysiotomy on the one side, and premature delivery and the application of forceps on the other. But he says that it will prevent the induction of labour too prematurely. The fear of an obstacle from the contracted pelvic cavity will no longer haunt us; and knowing that we have the power of enlarging the passage, we can wait calmly for the maturing of the fœtus, so that its capability for extra-uterine life may be certain. Symphysiotomy ought also to make all forcible applications of forceps disappear. Prof. Pinard adds that when a perfect application of the forceps has not, after moderate tractions, effected delivery, there is only one thing to be done; and that is, to withdraw the instrument and practise symphysiotomy.

Dr. J. Edwin Michael thinks[2] that symphysiotomy might save the child in a face presentation with a fixed and jammed posterior chin, when, on account of the position of the head, a Cæsarian section could not be done.

Special attention should be given to a communication by Wehle on "The Scientific Basis of Symphysiotomy," of which a very full translated and illustrated abstract is given in *The American Journal of Obstetrics* for May, 1893. He maintains that the division of the symphysis is always easy, and that a wide separation of the joint can be made without injury to the sacro-iliac synchondrosis. He also asserts that the subsequent ill effects of the operation can be reduced to almost *nil*.

[1] The lecture, translated by Dr. J. B. Henderson, is published in full in the *Lancet*, Feb. 18, 1893. [2] *Amer. Journ. Obst.*, Feb., 1893.

Much distress and suffering during the lying-in period often accompanies the process of nursing. For anything which can relieve this, both mother and attendants will be grateful.

Dr. Albert H. Ely,[1] writing on the inflammatory affections of the breast during lactation, points out that the exceptionally complex tissue of the breast renders it prone to inflammatory lesions, upon the proper treatment of which much depends, as there now exists strong evidence that a large percentage of the primary carcinomata of the breast are the result of mastitis. Attention should in all cases be particularly given to the nipple, as affections of this predispose to inflammations of the gland when any exciting cause presents. Lesions of the nipple may be divided into erosions, fissures of the apex, and fissures of the base. Dr. Ely insists on the importance of prophylaxis against these forms of sore nipples. No clothing at any time must be worn which will cause depression of the nipples. If this has occurred, and pregnancy exists, immediate efforts should be made to draw them out, and *eau de Cologne* or alcohol used during the early months as a lotion, and an alum solution in the last month. Even when no lesion exists, absolute cleanliness in nursing for mother and child must be insisted on, although it will probably be unnecessary to follow the example of some Continental hospitals, where a solution of bichloride is applied to the breast after nursing, and the child's mouth is cleansed with a solution of boric acid.

When an excoriation exists, Dr. Ely recommends the discontinuance of nursing for a time; and if the breast becomes engorged with milk, the application of pressure and the use of a solution of nitrate of silver. For fissures of the apex he employs a shield, and uses the solid nitrate of silver. For fissures at the base he adds the dusting of a dry dressing with aristol or like powder. For such troubles I have found a one-in-eight ointment of balsam of Peru very serviceable.

Dr. Ely, wisely arguing from the analogy of the brute mammalia, in which engorgement of the mammary gland often occurs when the milk fails to be excreted, urges rest and non-interference in similar conditions in human beings, and would put an end to the pounding and the use of the breast-pump, so popular with fussy nurses. It is difficult to convince them that the milk will go on being secreted, no matter what the withdrawing power may be.

In incipient mastitis, Dr. Ely suggests the trial of abortive measures, carried out upon strict antiseptic principles. The affected breast should be scrubbed with soap and water, and special attention directed towards cleansing the nipple and areola; oil of turpentine is then used over the whole surface; this is followed in like way by ether, and finally the gland is washed with corrosive sublimate solution. All such applications

[1] *New York Med. Journ.*, Dec. 31, 1892.

as poultices, ointments, liniments, and active massage must be abolished.

If the attempts at aborting fail and the formation of an abscess with fluctuation be accomplished, immediate incision should be employed with every antiseptic precaution. For this Dr. Ely gives very elaborate instructions, which no doubt could easily be carried out in a hospital, but which are not easy in general practice. With full surgical anæsthesia, he recommends the careful exploration of the cavity of the abscess, in order to discover any recess, and at the same time counter-incisions should be freely made for the release of any pus which may be lurking in them.

Dr. Samuel L. Weber also pleads [1] for a more thorough dealing with mammary abscess than is customary. He makes one or more incisions from the centre to the circumference of the gland and through it, searching with the finger in every direction for pus or disintegrated tissue, and then, while the irrigator plays, scrapes out all the cavities and sinuses with a large sharp spoon. The curetting must be thoroughly done if prompt healing is desired. Moist dressing, according to the details he gives, is to be used. Dr. Weber says that by this plan the worst cases are cured in from eight to twelve days, whilst by the current method, healing is prolonged to many weeks and even months; and he claims that a chronically indurated breast—a predisposing cause of carcinoma—is avoided.

Dr. S. Marx believes [2] that all inflammations of the breast, excepting those produced by traumatic influences and improperly regulated nursing, are due to an infection specifically caused by the entrance of toxic matter by way of fissures and erosions of the nipple. He therefore urges that nothing should be worn which by pressure might inflict injury upon the nipple of a pregnant woman, that it should be protected with plenty of absorbent cotton, and that the meddling advice of the textbooks should be discarded. If the nipple is inverted, drawing it out three times a week by means of a breast-pump will be sufficient. When the nipples are tender and sore, no other measure is recommended than the application of a ten per cent. ichthyol-lanoline salve. Dr. Marx considers that the use of a corrosive sublimate or carbolic solution is injurious. When the nipple becomes fissured and eroded, he uses an ointment recommended by Oehren, and composed of four parts of ichthyol, five each of lanoline and glycerine, and one of olive oil. This soon checks the excruciating pains of suckling, and the fissures rapidly heal. Owing to its consistency, the salve, which is nonpoisonous, can be easily and thoroughly washed off. A five or ten per cent. cocaine solution, applied shortly before putting the child to the breast, will ensure perfect freedom from pain.

In cases of threatened mastitis, Dr. Marx, in common with

[1] *Amer. Journ. Obst.*, Jan., 1893. [2] *Med. Rec.*, Feb. 11, 1893.

many others, employs firm compression, but secures this by a binder instead of a roller bandage, which does not ensure uniform pressure. Absolute rest is insisted upon, and iced lead-water applications are regularly applied.

It would undoubtedly be a good thing if not only the nipples of a pregnant woman were inspected from time to time, but if an examination of a more general character could be undertaken as a matter of routine. An analysis of the urine during pregnancy is becoming more general, though happily negative in an overwhelming majority of cases. A renewed plea for much more than this is put forth by Dr. J. Milton Mabbott,[1] who considers that it is a false modesty on the part of the patient and an unpardonable amount of indifference on the part of the doctor which permits the willing neglect, in any case, of means which some time may save a life. Dr. Mabbott says the examination should be made in all possible cases soon after they come under professional care, and certainly without unnecessary delay if the woman has passed the eighth month. He says that its utility consists in the conversion of podalic or pelvic into cephalic presentations in single pregnancies, the discovery of other malpositions of the fœtus, of a misplaced uterus, a uterine fibroma or carcinoma, an ovarian cystoma, an ectopic gestation, pelvic deformities or placenta prævia. Even if any of the causes of dystocia or of danger can not be remedied at the time of examination, the practitioner is better prepared to render the required assistance at the time of labour.

Dr. Mabbott says that we adopt the policy of indifference, because we are unwilling to add to the already too great anxiety associated with child-bearing; but with much cogency he insists that the assurance that "Everything is as it should be" would repay most women for the annoyance of submitting to the examination, whereas the early discovery of anything wrong would be a distinct gain. He concludes a wise paper by an eloquent aspiration for the speedy advent of the day when every pregnant woman, rich and poor, will be under the care of a qualified physician, and methodical examination be as general as the inspection of a sore throat before treatment. This will involve the relegation of the midwife to, what Dr. Mabbott calls, her legitimate field of nursing.

L. M. Griffiths.

[1] *New York Med. Journ.* Ap. 15, 1893.

